# Effects of the experience of breastfeeding-friendly practices and breastfeeding intention and self-efficacy on breastfeeding behavior: a cohort study in Taiwan

**DOI:** 10.1186/s13006-022-00539-9

**Published:** 2023-01-18

**Authors:** Yu-Wen Wang, Ying-Ju Chang

**Affiliations:** 1 Office of Research and Development, National Tainan Junior College of Nursing, Tainan, Taiwan; 2 Institution of Allied Health Sciences & Department of Nursing, College of Medicine, National Cheng Kung University, Tainan, Taiwan; 3 Department of Nursing, National Cheng Kung University Hospital, Tainan, Taiwan

**Keywords:** Breastfeeding friendly practice, Self-efficacy, Intention, Breastfeeding behavior

## Abstract

**Background:**

Approximately 80% of births in Taiwan occurred in Baby-Friendly accredited facilities, although the trend of exclusively breastfeeding infants until 6 months of age has stagnated in the last ten years. To guide breastfeeding promotion interventions during postnatal stays and encourage mothers to continue breastfeeding for the first 6 months, factors associated with breastfeeding behaviors to 6 months post-delivery must be investigated. This study explored the relationships among breastfeeding intention, experience of breastfeeding-friendly practices, breastfeeding self-efficacy, and breastfeeding behavior at four and 6 months after childbirth.

**Methods:**

In this longitudinal cohort study, women who gave birth to healthy newborns at two Baby-Friendly hospitals in Taiwan and who had initiated breastfeeding were recruited two to 4 days after giving birth. Data were collected three to 5 days after childbirth during hospitalization and at one, two, four, and 6 months after childbirth using a self-developed questionnaire to measure breastfeeding intention and the experience of breastfeeding-friendly practices, and the traditional Chinese version of the Breastfeeding Self-Efficacy Scale-Short Form to measure self-efficacy.

**Results:**

A total of 155 women completed the questionnaires five times within 6 months. The determinants of exclusive breastfeeding at 4 months and any breastfeeding at 6 months were the intention to breastfeed for over 6 months; a higher score for the experience of breastfeeding-friendly practices; and a higher level of breastfeeding self-efficacy during that period. The experience of breastfeeding-friendly practices during hospitalization did not predict breastfeeding behavior at 4 and 6 months. Intending to breastfeed for less than 4 months and lower breastfeeding self-efficacy during the hospital stay were both associated with shorter breastfeeding durations of less than 6 months after childbirth.

**Conclusions:**

Longer intended duration of breastfeeding, sustaining breastfeeding-friendly practices after discharge, and maintenance of a higher level of breastfeeding self-efficacy were the determinants of breastfeeding at 4 and 6 months. Healthcare professionals in Taiwan must support breastfeeding-friendly practices and consider interactive interventions to promote continued breastfeeding at different stages during the first 6 months after childbirth on the basis of the mother’s breastfeeding plan and breastfeeding self-efficacy during their postnatal hospitalization.

## Background

The Baby-Friendly movement was launched by the World Health Organization (WHO) and United Nations International Children’s Emergency Fund in the 1990s to improve maternal and pediatric health over the course of 30 years [1]. In accordance with the global advocacy and recommendations on breastfeeding [1–4], Taiwan implemented breastfeeding promotion policies in the 1990s to address the low rate of breastfeeding and the increase in use of infant formula [1, 5]. According to statistics published by Taiwan’s Ministry of Health and Welfare, the rate of exclusive breastfeeding at 6 months in Taiwan was 46.2% in 2018, with the objective to reach the WHO goal of at least 50% exclusive breastfeeding for the first 6 months by 2025 and 70% by 2030 [5–7]. In 2020, 73.2% of infants were born in Baby-Friendly accredited facilities in Taiwan, yet only 10% of infants in the world were born in such facilities [4, 8]. Conversely, the impact of Baby-Friendly accredited facilities on breastfeeding is lower in the last ten years than before 2011 in Taiwan, and the rate of exclusive breastfeeding at 6 months decreased and then stagnated [5, 9]. To meet the WHO’s target of achieving a 70% exclusive breastfeeding rate by 2030, Taiwan must improve its breastfeeding promotion strategies.

Studies have investigated various associated factors of breastfeeding behavior, including demographic information such as the mother’s age, education level, and delivery type [10, 11], maternal psychosocial status such as breastfeeding intention and self-efficacy [12–14], and practice of the Ten Steps to Successful Breastfeeding [15]. The related factors of breastfeeding may also differ among countries because of their distinct economic conditions and sociocultural factors [16–18]. In Taiwan, to encourage and support breastfeeding, Baby-Friendly hospital practices were implemented in the labor, delivery, postpartum, and maternity hospitalization periods [19, 20]. However, to achieve the status of being a certified Baby-Friendly Hospital in Taiwan, hospitals strictly implemented Baby-Friendly practices without providing appropriate mother-friendly support, thus causing stress among Taiwanese mothers during their hospitalization [9, 21]. Hence, the implementation must be reviewed and updated in an innovative manner by considering women’s autonomy and the Taiwanese traditional culture of postpartum care [9, 21]. To this end, this study explored the influence of breastfeeding intention, experience of Baby-Friendly practices, and maternal breastfeeding self-efficacy within 6 months on breastfeeding outcomes at four and 6 months. The identification of determinants is useful for devising appropriate breastfeeding promotion programs in hospitals and communities to encourage mothers to continue breastfeeding for the first 6 months after childbirth.

This study examined the relationships among breastfeeding intention, experience of breastfeeding-friendly practices, breastfeeding self-efficacy, and breastfeeding behavior at different time points within 6 months after childbirth. The secondary aim was to identify the determinants of breastfeeding behaviors at four and 6 months and baseline predictors of breastfeeding duration within 6 months after childbirth.

## Methods

### Study design and participants

A longitudinal cohort study was conducted over 6 months after childbirth from September 2019 to August 2020. Experience of breastfeeding-friendly practices and breastfeeding self-efficacy were measured using self-reported questionnaires three to 5 days after giving birth in the postpartum wards and at one, two, four and 6 months after childbirth. This study consecutively recruited women 20 years or older who gave birth to healthy newborns at a gestational age of 36 to 42 weeks at two Baby-Friendly-accredited hospitals in Taiwan; had initiated breastfeeding; and could read or write Traditional Chinese. The exclusion criteria were as follows: (1) abnormal maternal or neonatal health conditions after delivery that required hospital admission; (2) prohibition of breastfeeding according to medical recommendation; and (3) multiple births. The sample size was estimated with the rule of thumb: logistic regression and Cox models should be used with a minimum of ten outcome events for each predictor variable [22]. The planned attrition rate was calculated at 20% according to related studies [14], and the estimated sample size was 170.

### Instruments

The instruments consisted of four self-reported questionnaires on maternal characteristics, the experience of breastfeeding-friendly practices, breastfeeding self-efficacy, and breastfeeding behavior. The maternal characteristics, including the participant’s age, education level, marital status, occupation before childbirth, time of return to work, occupation after childbirth, parity, delivery type, experience of breastfeeding, and breastfeeding intention, were recorded using a self-developed questionnaire. Breastfeeding intention was defined as the duration for which the mother was willing to breastfeed [14], and the responses for breastfeeding intention were grouped into 1–4, 4–6, 6–12, and >  12 months [9, 14]. The range of the item content validity index (I-CVI) was 0.9–1; the average content validity index (S-CVI) of scales was 0.99.

The experience of breastfeeding-friendly practices in this study was defined as the mother’s experience of the Baby-Friendly practices implemented directly for mothers or infants to support breastfeeding and was measured using a seven-item self-developed questionnaire. In reference to the WHO Ten Steps to Successful Breastfeeding in 2018 [4], the experience of breastfeeding-friendly practices in this study was categorized into the experience of “immediate postnatal care” during maternity hospitalization and “breastfeeding establishment and infant feeding practices” from maternity hospitalization to 6 months after childbirth. Immediate postnatal care comprised the two items of skin-to-skin contact (categorized as Yes and No) and timing of breastfeeding initiation (categorized as < 1, 1–4, 4–24, and >  24 hours). The second section on the experience of breastfeeding-friendly practices was composed of five items that measured breastfeeding establishment and infant feeding practices on a 5-point Likert scale for (1 = never, 2 = rarely, 3 = sometimes, 4 = often, and 5 = always); and the total score of the experience of breastfeeding-friendly practices was 25 points. Items for breastfeeding establishment and infant feeding practices included the experience of rooming-in, maintaining the production of breast milk, breastfeeding on demand, avoiding feeding bottles and teats, and avoiding the use of pacifiers. The S-CVI of the experience of breastfeeding-friendly practices during hospitalization was 1, and that at one, two, four and 6 months was 0.96.

Breastfeeding self-efficacy was defined as a mother’s perceived confidence in her ability to breastfeed her infant [23] and was measured using the traditional Chinese version of the Breastfeeding Self-Efficacy Scale-Short Form (BSES-SF), as translated by Hu [24]. The original Breastfeeding Self-Efficacy Scale (BSES) was developed by Dennis and Faux in 1999 and is based on breastfeeding self-efficacy theory. In 2003, Dennis revised the original 33-item BSES into the 14-item BSES-SF, and psychometrically, the revised BSES-SF was divided into two domains, namely technical and intrapersonal thoughts [25]. The BSES-SF employed a 5-point Likert scale, with the score of each item ranging from 1 (not at all confident) to 5 (always confident). The total BSES-SF score ranged from 14 to 70, with a higher score representing a higher level of maternal self-efficacy in breastfeeding. Cronbach’s alpha was 0.94 for the original BSES-SF (*n* = 667) [25] and 0.93 (*n* = 48) for the traditional Chinese version of the 14-item BSES-SF [24]. In this study, Cronbach’s alpha for the traditional Chinese version BSES-SF was 0.95 (*n* = 155).

Breastfeeding behavior was assessed using a structured questionnaire developed by the authors. Based on the definition of breastfeeding indicators by the WHO in 2008 and standardized indicators of breastfeeding described by Greiner in 2014, breastfeeding behavior was measured using point-in-time data (24-hour recall) and data reflecting breastfeeding behaviors since birth [26, 27]. The indicators of breastfeeding behavior in this study included feeding patterns and the duration of breastfeeding, and three types of breastfeeding patterns were referenced according to what the infant was fed in the past 24 hours [27, 28]. Exclusive breastfeeding indicated that the infant was fed no other food or drink, not even water, other than breast milk, although they may have received prescribed medicines, oral rehydration solutions, or vitamins and minerals [28]. Partial breastfeeding was defined as the feeding pattern in which the infant receives breast milk supplemented with baby formula or solid or semisolid foods. Replacement feeding referred to the feeding pattern in which the infant receives baby formula milk or solid or semisolid foods without breast milk [26, 29]. Exclusive breastfeeding and partial breastfeeding were grouped under “any breastfeeding.” Breastfeeding duration was defined as the length (in weeks) of any breastfeeding within 6 months after childbirth. Both the I-CVI and S-CVI calculated by five experts of maternal nursing and instrument development were 1.

### Data collection and study procedure

After approval to conduct the study was obtained from the institutional review boards of the two participating institutions, eligible women were recruited from these hospitals. Women who met the eligibility criteria were approached by the researcher two to 4 days after giving birth in the respective maternity wards. The study participants provided informed consent after a verbal explanation of the study procedure and objectives was provided. An online survey system was used to deliver the questionnaire through e-mail or mobile instant messaging applications during participant hospitalization and at the follow-ups of one, two, four and 6 months after childbirth. In Taiwan’s National Health Insurance program, women can receive 3 days of hospital care after vaginal birth and 5 days after a Cesarean section. Therefore, we conducted the first survey three to 5 days after childbirth, depending on the participant’s length of stay. The first survey comprised questions on maternal characteristics, experience of breastfeeding-friendly practices, breastfeeding self-efficacy, and breastfeeding behavior. Follow-ups at one, two, four and 6 months after childbirth were conducted for data collection on the experience of breastfeeding-friendly practices, breastfeeding self-efficacy, and breastfeeding behavior.

### Data analysis

Statistical analysis was performed using R software v4.0.5, and descriptive statistics were obtained to explain the demographic data. Factors associated with breastfeeding behaviors in different periods after childbirth were examined using one-way analysis of variance (ANOVA) and the Kruskal–Wallis, Chi-square, Fisher’s exact test, and Wilcoxon rank sum tests based on the data type of the variables. Multivariable multinomial logistic regression and multiple logistic regression were conducted to identify the determinants of breastfeeding behavior at four and 6 months after childbirth. In addition, Cox regression analyses were performed to identify factors associated with breastfeeding duration; a nomogram measuring the probability of breastfeeding termination at 6 months was also generated. A *P*-value of less than 0.05 represented the level of significance.

## Results

A total of 170 participants were recruited into this study, of which 155 completed the questionnaires five times over the course of 6 months, representing an attrition rate of 9%. The majority of the women were married, had college degrees, full-time jobs before childbirth, and delivered vaginally. Approximately half of the women were first-time mothers. None of the women intended to breastfeed their infants for less than 1 month. Most of the women (71.0%) had skin-to-skin contact occurred immediately after childbirth in the delivery room, but none of the women initiated breastfeeding within the first hour of childbirth (Table 1). No significant difference in maternal and neonatal characteristics was noted between the two hospitals.

**Table 1:** Maternal characteristics of the participants (*N* = 155)

Variables		N(%) / mean ± SD
**Age (years)**		35 ± 5
**Education**	Senior high or below	13 (8.4)
	College	106 (68.4)
	Graduate school or above	36 (23.2)
**Marital Status**	Married and living with spouse	154 (99.4)
	Unmarried	1 (0.6)
**Occupation before childbirth**	Full-time job	114 (73.5)
	Homemaker	35 (22.6)
	Part-time job	5 (3.2)
	Student	1 (0.6)
**Return to work**	< 2 months after childbirth	43 (27.7)
	2–6 months after childbirth	23 (21.3)
	> 6 months after childbirth	48 (31.0)
	Not employed	31 (20.0)
**Occupation after childbirth**	Full-time job	85 (54.8)
	Homemaker	32 (20.6)
	Part-time job	3 (1.9)
	Parental leave	35 (22.6)
**Parity**	1	81 (52.3)
	2	58 (37.4)
	≥ 3	16 (10.3)
**Mode of birth**	Vaginal	84 (54.2)
	Cesarean	71 (45.8)
**Experience of breastfeeding**	Yes	74 (47.7)
**Breastfeeding intention**	1–4 months	22 (14.1)
	4–6 months	41 (26.5)
	6–12 months	55 (35.5)
	> 12 months	37 (23.9)
**Skin-to-skin contact**	Yes	110 (71.0)
**Timing of breastfeeding initiation**	< 1 hour	0 (0)
	1–4 hours	54 (34.8)
	5–24 hours	62 (40.0)
	> 24 hours	39 (25.2)

*SD* Standard deviation

**Fig. 1:**
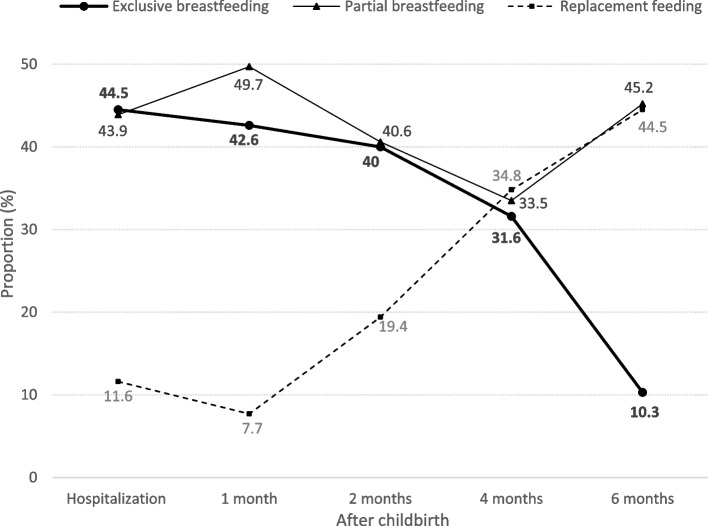
Feeding patterns within 6 months of childbirth (*N* = 155)

All the participants began breastfeeding within 48 hours of childbirth during hospitalization. In terms of breastfeeding patterns, the rate of exclusive breastfeeding was 44.5% (*n* = 69) at 3–5 days after child birth during hospitalization and 31.6% (*n* = 49) and 10.3% (*n* = 16) at four and 6 months after childbirth, respectively. The rate of partial breastfeeding was 43.9% (*n* = 68) at three to 5 days after childbirth during hospitalization and 33.5% (*n* = 52) and 45.2% (*n* = 70) at four- and six-months after childbirth, respectively (Fig. 1). Thirty-five women (22.6%) exclusively breastfed their infants for the first 4 months and then switched to partial breastfeeding because they introduced supplementary food (including solid or semisolid foods) with breastfeeding from four to 6 months. Table 2 lists the associated factors of breastfeeding patterns at each time point after childbirth. At each time point, longer intended duration of breastfeeding, higher scores for the experience of breastfeeding-friendly practice, and higher level of breastfeeding self-efficacy were all positively associated with exclusive breastfeeding. Maternal occupation, the timing of return to work, and parity were not associated with the three breastfeeding patterns at any time point after childbirth (Table 2).

**Table 2:** Association between factors and breastfeeding patterns at different follow-up times after childbirth (*N* = 155)

	Feeding patterns Exclusive BF/ Partial BF/ Replacement feeding ***p***				
	Hospitalization	1st month	2nd month	4th month	6th month
**Factors recorded during hospitalization**					
**Age**^**a**^	0.057	0.009**	0.019 *	0.374	0.950
**Education**^**c, d**^	0.849	0.017*	0.214	0.050	0.750
**Delivery Type**^**d**^	0.300	0.019*	0.528	0.649	0.903
**BF intention**^**c**^	0.026*	0.0006***	0.0005***	0.0005***	0.0005***
**Time to initiate BF**^**c, d**^	0.006**	0.129	0.013*	0.021*	0.070
**Factors recorded in different periods**					
**Experience of BF friendly practices**^**a, b**^	< 0.0001***	< 0.001***	< 0.0001***	< 0.0001***	< 0.0001***
**BF self-efficacy**^**a, b**^	< 0.0001***	< 0.0001***	< 0.0001***	< 0.0001***	< 0.0001***

^a^Analyzed using one-way ANOVA

^b^Analyzed using the Kruskal–Wallis test

^c^Analyzed using the Chi-square test;

^d^Analyzed using Fisher’s exact test in an r × c table in R software v4.0.5 [30]

*BF* Breastfeeding

*p < 0.05; ** *p* < 0.01, *** *p* < 0.001

To identify the determinants of breastfeeding behavior at four- and six-month follow-up, variables that exhibited a significant association with feeding patterns at these time points were included in multivariable multinomial logistic regression models. The participants who began breastfeeding within 5–24 hours of giving birth (adjusted odds ratio [OR] = 4.35; 95% confidence interval [CI]: 1.42, 13.58) and had a higher total self-efficacy score (adjusted OR 1.12; 95% CI: 1.07, 1.19) at 4 months were more likely to exclusively breastfeed than partially breastfeed at 4 months postpartum. Mothers with the intention to breastfeed for over 6 months (adjusted OR = 14.3 and 20; 95% CI: 1.09, 180.54 and 1.14, 344.30) and who had a higher total score of the experience of breastfeeding-friendly practices (adjusted OR 1.35; 95% CI: 1.06, 1.70) and self-efficacy at 4 months (adjusted OR 1.15; 95% CI: 1.08, 1.23) were more likely to exclusively breastfeed than provide food or formula feeding at 4 months postpartum (Table 3).

**Table 3:** Determinants of exclusive breastfeeding at 4 months (*N* = 155)

		Exclusive BF		Exclusive BF	
		vs. Partial BF		vs. Replacement feeding	
		Adjusted OR	95% CI	Adjusted OR	95% CI
**BF intention**	4–6 months	2.32	(0.17, 31.73)	8.33	(0.59, 123.19)
	6–12 months	1.96	(0.17, 22.67)	14.29*	(1.09, 180.54)
	≥ 12 months	2.13	(0.17, 26.55)	20.00*	(1.14, 344.30)
	Reference: 1–4 months				
**BF initiation**	5–24 hours	4.35*	(1.42, 13.58)	2.22	(0.47, 10.56)
	> 24 hours	1.61	(0.40, 6.49)	0.50	(0.08, 2.94)
	Reference: 1–4 hours				
**Experience of BF friendly practices at 4 months**		0.95	(0.80, 1.13)	1.35**	(1.06, 1.70)
**BF self-efficacy at 4 months**		1.12**	(1.07, 1.19)	1.15**	(1.08, 1.23)

Analyzed through multivariable multinomial logistic regression

*BF* Breastfeeding, *CI* Confidence interval: *OR* Odds ratio

*p < 0.05; ** *p* < 0.01

Because of the considerable reduction in the exclusive breastfeeding rate and the unbalanced distribution of participants in terms of the breastfeeding patterns at 6 months, logistic regression models were applied to identify the determinants of any breastfeeding at 6 months. Mothers with the intention to breastfeed for over 6 months (adjusted OR 0.04; 95% CI: 0.00, 0.30) and who had a higher score for the experience of breastfeeding-friendly practices (adjusted OR 0.69; 95% CI: 0.53, 0.84) and self-efficacy at 6 months (adjusted OR 0.94; 95% CI: 0.89, 0.98) were less likely to replace any breastfeeding with alternative feeding at 6 months postpartum (Table 4).

**Table 4:** Determinants of any breastfeeding at 6 months (*N* = 155)

	Estimate	SE	Z	Adjusted OR	95% CI
**(Intercept)**	10.95	2.09	5.23***		
**BF intention Reference:** 1–4 **months**					
**4–6 months**	−2.19	1.15	−1.90	0.11	(0.01, 0.90)
**6–12 months**	−3.24	1.14	−2.83**	0.04	(0.00, 0.30)
**≥ 12 months**	−4.58	1.36	−3.38***	0.01	(0.00, 0.11)
**Experience of BF friendly practices at 6 months**	−0.38	0.11	−3.30***	0.69	(0.53, 0.84)
**BF self-efficacy at 6 months**	−0.06	0.02	−2.60**	0.94	(0.89, 0.98)

Analyzed through multiple logistic regression

*BF* Breastfeeding, *CI* Confidence interval, *OR* Odds ratio, *SE* Standard error

**p < .01; *** *p* < .001

Eighty-six participants (55.5%) in this study continued breastfeeding their infants after 6 months of childbirth. The mean duration of breastfeeding in the women who discontinued breastfeeding before the first 6 months of childbirth (*n* = 69, 44.5%) was 10.4 ± 6.6 weeks (ranging from 1 to 25 weeks). Univariate analysis of the Cox proportional hazard model revealed that parity, intention to breastfeed, time of initiation of breastfeeding, and in-hospital breastfeeding self-efficacy were associated with breastfeeding duration within 6 months after childbirth. The baseline predictors of breastfeeding duration within 6 months were next analyzed through multivariate Cox regression analysis. Participants who expressed an intention to breastfeed for four to 6 months had a 60% lower risk (hazard ratio [HR] 0.40; 95% CI: 0.22, 0.71) of ceasing breastfeeding at any time point compared with those who intended to breastfeed for one to 4 months following adjustment for confounders. Women with the intention to breastfeed for 6–12 months and more than 12 months had 86% (HR 0.14; 95% CI: 0.07, 0.27) and 92% (HR 0.08; 95% CI: 0.03, 0.21) lower risks of ceasing breastfeeding at any time point compared with women who intended to breastfeed for 1–4 months after adjustment for confounders. The HR of breastfeeding termination within 6 months was significantly lower when participants had a higher level of breastfeeding self-efficacy during hospitalization (HR 0.98; 95% CI: 0.96, 1.00) (Table 5).

**Table 5:** Multivariable Cox proportional hazard model for breastfeeding duration at 6 months (*N* = 155)

	Coefficient	HR	95% CI	Z
**BF intention Reference:** 1–4 **months**				
**4–6 months**	− 0.92	0.40***	**(0.22, 0.71)**	− 3.10
**6–12 months**	−1.97	0.14***	**(0.07, 0.27)**	−5.78
**≥ 12 months**	−2.51	0.08***	**(0.03, 0.21)**	−5.14
**BF self-efficacy during hospitalization**	− 0.02	0.98***	**(0.96, 1.00)**	−2.05

*BF* Breastfeeding, *CI* Confidence interval, *HR* Hazard ratio

*** *p* < .001

**Fig. 2:**
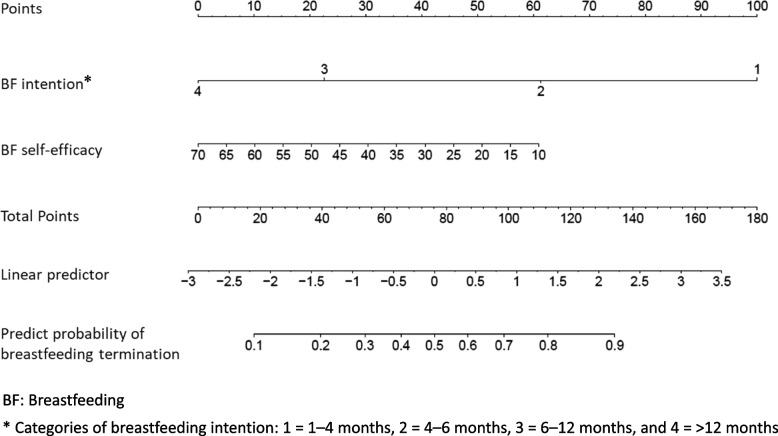
Nomogram for the probability of breastfeeding termination within 6 months

To develop and validate the nomogram for predicting the probability of breastfeeding termination within 6 months from maternal hospitalization, the predictors of breastfeeding duration at 6 months were included in the nomogram. The data of all participants were separated into the training dataset (*n* = 125) and validation dataset (*n* = 30). Logistic regression was applied to construct the prediction nomogram according to the variables of breastfeeding intention and breastfeeding self-efficacy in the training dataset. The validation dataset was used to test and evaluate the training model; from the confusion matrix, accuracy, specificity, and sensitivity were 0.76, 0.86, and 0.69, respectively. To obtain the prediction of the probability of breastfeeding termination within 6 months, participants’ values at each axis were first identified, and the sum of the two scores was plotted on the Total Points line to evaluate the probability of breastfeeding termination on the “Predict the probability of breastfeeding termination” line, as illustrated in Fig. 2. For example, for a woman intending to breastfeed for 6–12 months (categorized in Group 3, 23 points) with a total score of 30 for breastfeeding self-efficacy (41 points), the sum was 64 points, and the probability of breastfeeding termination within 6 months was approximately 39% (Fig. 2). The predictive ability of the nomogram was evaluated through analysis of the receiver operating characteristic with an area under the curve of 0.87 and cut-off point of 0.65 (specificity = 0.88 and sensitivity = 0.79).

## Discussion

This study revealed the determinants of breastfeeding within 6 months after childbirth in Taiwan. The cohort of women demonstrated a low prevalence of exclusive breastfeeding and a high prevalence of partial breastfeeding in the 6 months after childbirth, which is consistent with the findings of other studies in Taiwan [14] and those in other high-income countries [16, 31]. Exclusive breastfeeding within 6 months in this study exhibited a sharp decrease from 31.6% at 4 months to 10.3% at 6 months. Similar results were reported in a Taiwanese study surveying 300 primiparous mothers [14]. Regarding the introduction of supplementary food, the WHO and American Academy of Pediatrics have advised that infants be exclusively breastfed for the first 6 months, with continued breastfeeding alongside the introduction of suitable complementary foods up to 2 years of age or beyond [2, 28]. The Taiwan Pediatric Association (2016) recommends that infants be exclusively breastfed for the first four to 6 months, with complementary foods introduced at four to 6 months [32]. This recommendation may have affected the marked shift in the feeding pattern from exclusive to partial breastfeeding.

The association between demographic attributes and exclusive breastfeeding was limited to within 2 months after childbirth with respect to the mothers’ college education level, younger age, and multipara status. These results are partially consistent with those of another Taiwanese study [33]. However, in previous studies, returning to the workplace after childbirth may have influenced the effectiveness of breastfeeding promotion programs within 6 months postpartum in Taiwan [33, 34]. In the current study, the occupational type and time of return to work were not associated with breastfeeding behavior at any time point within 6 months, possibly due to the effectiveness of Taiwan’s enforcement of laws and regulations regarding women’s employment rights in the last five to ten years. In 2016, amendments to the provisions for parental leave in the Act of Gender Equality in Employment may have affected the relationship between employment and women’s breastfeeding behavior within the first 6 months postpartum [35].

The higher scores for the experience of breastfeeding-friendly practices were attributable to the determinants of exclusive breastfeeding at 4 months and any breastfeeding at 6 months in this study, although the association between the experience of immediate postnatal care (including skin-to-skin contact and initiation of breastfeeding within 4 hours) and breastfeeding behavior did not extend to four to 6 months postpartum. Breastfeeding early after birth can be physiologically enabled through physical closeness of the mother and infant, skin-to-skin contact immediately after childbirth, ongoing rooming-in, and feeding of the infants on demand [36]. In contrast to the common perception of the positive effect of immediate postnatal care on breastfeeding behavior [37], none of the women who had skin-to-skin contact initiated breastfeeding in the first hour after birth, and the women who began breastfeeding within 5–24 hours of giving birth were more likely to breastfeed exclusively at 4 months compared with those who began breastfeeding within one to 4 hours after giving birth in this study. One study reported that 7.1% of Taiwanese women initiated breastfeeding in the first hour after childbirth [14]. The study also indicated that initiating breastfeeding within one to 4 hours of childbirth only contributed to exclusive breastfeeding during hospitalization after delivery, with limited effects on breastfeeding at one and 6 months [14]. The unexpected result that no women initiated breastfeeding during skin-to-skin contact in this study might be attributable to the lack of information regardingthe benefits of early breastfeeding initiation and barriers to skin-to-skin contact from health-care professionals. In Taiwan, the excessive workload of health-care professionals and lack of promotion of early breastfeeding initiation when performing skin-to-skin contact; the traditional practices of postpartum confinement, including the advice provided to women to rest to facilitate recovery in the first month after childbirth; fatigue caused by the aggressive implementation of Baby-Friendly practices during hospitalization without the provision of suitable support to Taiwanese mothers; and the shortage of professional health-care support for mother-friendly childbirth may have weakened the positive effects of immediate postnatal care on long-term breastfeeding [9, 14, 20, 38, 39].

The intention to breastfeed for over 6 months already decided during hospitalization and a higher level of breastfeeding self-efficacy were key determinants in mothers who breastfed their infants at the four- and six-month follow-up. In-hospital breastfeeding self-efficacy is also a critical predictor of breastfeeding behaviour found by many other researchers [12–14, 40]. This study verified and extended the results of other studies indicating that the maintenance of a higher level of breastfeeding self-efficacy was a crucial determinant at 4 months of exclusive breastfeeding and at 6 months of any breastfeeding. Therefore, more specific and stronger interventions are required in Taiwan based on the status of breastfeeding self-efficacy to encourage breastfeeding exclusively for the first 6 months postpartum. Further research is warranted to assist in the implementation of appropriate interventions, which should incorporate the multifaceted factors of breastfeeding at each time point in the first 6 months to support successful breastfeeding.

This study had some limitations. Employing convenience sampling, recruiting participants from a single region in Taiwan, and including two hospitals in a hierarchical medical system may have limited the generalizability of these findings. Another limitation is recruiting postpartum women because breastfeeding intention is better measured prior to birth [9, 12]. Data on skin-to-skin contact and breastfeeding initiation were recorded using a self-reported questionnaire; therefore, the infants’ readiness to feed with skin-to-skin contact and how much hospital staff encourage breastfeeding within the first hour of childbirth remain unknown. In addition, breastfeeding behavior was recorded through 24-hour recalls; thus, self-reporting and social desirability may bias the result.

### Implications for practice

Breastfeeding intention and self-efficacy were the major baseline predictors of breastfeeding duration within the first 6 months postpartum, reflecting similar results of studies in Taiwan and other high-income countries [9, 13, 14, 41]. Maternal and pediatric health care professionals must assess the probability of breastfeeding termination within these initial 6 months, assess breastfeeding self-efficacy repeatedly after a mother’s discharge, recognize the main factors affecting breastfeeding at each stage, and support the extension of breastfeeding duration up to 6 months according to the factors at each stage. Breastfeeding intention and breastfeeding self-efficacy during hospitalization could be used to pre-estimate breastfeeding behavior in the initial 6 months after delivery, and health care professionals must provide breastfeeding-friendly practices and educational interventions to promote breastfeeding at different stages within these 6 months based on these pre-estimates. Discharge support and individualized interactive educational interventions for breastfeeding are also required to address each family’s particular needs and ensure the provision of effective breastfeeding-friendly practices [42].

## Conclusions

This study demonstrated the influence of breastfeeding intention, the experience of breastfeeding-friendly practices, and breastfeeding self-efficacy on breastfeeding behavior within 6 months after childbirth in Taiwan. With the universalization of Baby-Friendly-accredited institutions and the popularization of breastfeeding-friendly practices in Taiwan, the continuation of the implementation of breastfeeding-friendly practices after discharge and maintenance of improved breastfeeding self-efficacy are key factors at four to 6 months for exclusive breastfeeding and any breastfeeding. The findings of this study indicated that breastfeeding intention and self-efficacy can be used to pre-estimate breastfeeding behavior within 6 months postpartum. Breastfeeding promotion policies and Baby-Friendly practices have been implemented in Taiwan for 30 years. In response to ongoing societal changes, the government and health care systems must continue to reassess and regulate breastfeeding promotion policies. To achieve the WHO’s goals of encouraging exclusive breastfeeding and realizing long-term benefits of mother-child health, health-care professionals must provide more information antenatally in order to normalize breastfeeding and increase women’s intended breastfeeding duration; breastfeeding-friendly interventions; lactation management services to support breastfeeding at various stages after childbirth on the basis of individual’s pre-estimation results and mothers’ plans; and community education on breastfeeding [17, 43].

## Data Availability

Data cannot be made publicly available in order to protect the privacy of mothers and infants.

## Abbreviations

ANOVA: Analysis of Variance
BF: Breastfeeding
BSES: Breastfeeding Self-Efficacy Scale
BSES-SF: Breastfeeding Self-Efficacy Scale-Short Form
CI: Confidence interval
HR: Hazard ratio
I-CVI: Item content validity index
OR: Odds ratio
S-CVI: Average content validity index
SD: Standard deviation
SE: Standard error
WHO: World Health Organization
